# Mechanism of Double-Diffusive Convection on Peristaltic Transport of Thermally Radiative Williamson Nanomaterials with Slip Boundaries and Induced Magnetic Field: A Bio-Nanoengineering Model

**DOI:** 10.3390/nano13050941

**Published:** 2023-03-05

**Authors:** Safia Akram, Maria Athar, Khalid Saeed, Alia Razia, Taseer Muhammad, Huda Ahmed Alghamdi

**Affiliations:** 1 Military College of Signals (MCS), National University of Sciences and Technology, Islamabad 44000, Pakistan; 2 Department of Mathematics, National University of Modern Languages, Islamabad 44000, Pakistan; 3 Department of Mathematics, COMSATS University, Islamabad 44000, Pakistan; 4 Department of Mathematics, College of Science, King Khalid University, Abha 61413, Saudi Arabia; 5 Department of Biology, College of Science, King Khalid University, Abha 61413, Saudi Arabia

**Keywords:** thermal radiation, induced magnetic field, viscous dissipation, double-diffusive convection, slip boundaries, Williamson nanofluid, asymmetric channel

## Abstract

The present work has mathematically modeled the peristaltic flow in nanofluid by using thermal radiation, induced a magnetic field, double-diffusive convection, and slip boundary conditions in an asymmetric channel. Peristalsis propagates the flow in an asymmetric channel. Using the linear mathematical link, the rheological equations are translated from fixed to wave frames. Next, the rheological equations are converted to nondimensional forms with the help of dimensionless variables. Further, the flow evaluation is determined under two scientific assumptions: a finite Reynolds number and a long wavelength. Mathematica software is used to solve the numerical value of rheological equations. Lastly, the impact of prominent hydromechanical parameters on trapping, velocity, concentration, magnetic force function, nanoparticle volume fraction, temperature, pressure gradient, and pressure rise are evaluated graphically.

## 1. Introduction

The field of thermal radiation gains interest of researchers owing to its multifarious utility in manufacturing industry, engineering, and mechanics. In mechanical manufacturing, the process is involved in nuclear technology, manufacturing of glass sheets, cooling procedures of high heating mechanisms, boiler designs, cooling methods for gases, etc. In the phenomenon, heat is exchanged through electromagnetic waves in the form of radiation. Hence, most of the energy conversion procedures that run at a high temperature involve thermal radiation in their heat transfer mechanisms. Thermal radiation is vital in the procedures that have large temperature differences between the surface area and the surrounding region. Numerous studies [1,2] have focused on the thermal linear radiation impact on the heat exchange of non-Newtonian and Newtonian liquids on expanded surfaces. However, the method of linear radiation is no more effective in indefinite parameters, and the procedures involved considerable temperature differences, like those needed for the linear Rosseland estimation [3]. On the other hand, the nonlinear estimation involved three types of parameters: the radiation, the temperature ratio, and the Prandtl number. The impact of the three parameters, namely nonlinear radiation, heat target, and predisposed magnetic flux, on nanofluid’s flow was studied by Hayat [4]. Khan et al. [5] elaborated on gyrotactic microorganisms and nonlinear thermal radiation’s influence on a magnetized Burgers’ nanofluid. In Moshizi and Malvandi’s study [6], the various procedures of nanoparticle shift on the mixed convection of ${\mathrm{Al}}_{2}O_{3}$, where water nanofluid motion in an upright micro annulus was discussed.

Nowadays, the research trend has shifted toward the examination of non-Newtonian peristaltic flow, which has wide applications in environmental, geophysical, industrial, and engineering domains. In human anatomy, the phenomenon of peristalsis is a driving force. Natural fluid transmission in the human body, such as food ingestion and digestion in gastrointestinal pathways, the excretion of wastes from the body, blood circulation, and the secretion of enzymes, hormones, etc. involves peristalsis. The phenomenon was introduced by Latham [7] in mathematics. Later, various investigations have been conducted to explore and analyze the phenomenon under different conditions and with varying parameters, such as wavelength and amplitude. Sucharitha et al. [8] explored the phenomenon in a permeable medium for non-Newtonian fluids. Similarly, Srivastava and Saxena [9] applied the two-fluid model of non-Newtonian blood flow instigated by peristalsis. Later, the phenomenon was combined with magnetohydrodynamic (MHD) flow of a fluid in a channel with reference to the physiological flow of fluids, such as blood transportation and transfusion processes. On theoretical grounds as well as practical grounds, this also raises interest in future research, which could involve peristaltic MHD compressor operation. In this regard, Sud et al. [10] investigated the behavior of blood flow under dynamic magnetic field. Agrawal and Anwaruddin [11] constructed a mathematical model for MHD blood flow within multilevel channels under flexible boundaries by applying a peristalsis wave with a long wavelength. With the help of this model and by using the estimation technique, they observed the blood flow in diseased arteries as having arteriosclerosis and stenosis. They suggested that applying magnetic force may be used as a blood pump during cardiac operations. The magnetic-field principle is used in magnetic resonance imaging (MRI) for patients under high constant magnetic flux. Recent investigations into Newtonian and non-Newtonian fluids under magnetic force and different flow geometries are listed in [12,13,14,15,16,17].

The slip condition applied in combination with this phenomenon has produced marvelous results. The features of flowing fluid under boundary conditions have a significant relationship with many applications. Kwang et al. [18] studied peristalsis in non-Newtonian fluid by applying the slip condition in a 2D microchannel. Hence, the fluids must be under slip conditions to have notable applications in the bioindustry. Artificial heart polishing valve is the common example. Bhatti and Sara [19] explored the Hall and Ion slip impact on non-Newtonian nanofluid, which was kept in an uneven medium under peristalsis flow. Mekheimer et al. [20] applied the peristalsis and a suspension slip flow in a rectangular duct with lateral walls. Many researchers have further explored the partial slip impact on non-Newtonian fluids, as given in [21,22,23,24,25].

Numerous researchers are actively engaged in exploring the domain of nanofluids thanks to its noteworthy utilization in biotech engineering, mechanical and manufacturing industries, and the automotive industry. Liquids that have nanoparticles equally distributed throughout the basic fluid, such as water, ethylene, and oil, are known as nanofluids [26]. Experimental data have revealed that the nanofluids have a unique property of augmenting heating properties when they are mixed with other liquids and substances [27,28,29]. The common methods: the Tiwari-Das model [30] and the Buongiorno model [31] are widely used in studies. The first one [30] revolves around the volume fractions of nanoparticles, while the second method [31] considers the impact of thermophoresis, along with Brownian flow. Moreover, the Buongiorno model is more inclined toward the observation of heat intensification under convection, but there is some modification applied in the model to incorporate the thermophysical characteristics of nanoparticles [32]. In this regard, Tripathi and Beg [33] numerically analyzed nanofluid peristaltic flow and also observed the drug-delivery efficiency. The same study was conducted by Awais et al. [34], in this case applying a hybrid model. Similarly, Bibi and Xu [35] examined the chemical changes during peristaltic nanofluid flow by using a hybrid model. Other significant studies on the phenomenon are given in [36,37,38,39,40,41,42,43].

Another important physical concept, double-diffusive convection, is characterized as the two density ingredients fused at differing rates [44]. Narayana et al. [45] studied the phenomenon by applying consistent heating and salt on a curved surface in a permeable container. Likewise, Siddiqa et al. [46] examined double-diffusive-free convection in a nonabsorbent container. Further to this, Ibrahim and Marin [47] used pulse laser heating to obtain a numerical solution of thermoelastic interactivity in a half-vacuumed area. Prasad et al. [48] applied magnetic flux and thermal diffusion to observe mixed convection flow on an expedite erect curved plate in a permeable chamber. More work on double-diffusive convection on peristaltic nanofluid flow is mentioned in references [49,50,51,52,53,54,55].

The phenomenon of nanofluid peristalsis with double-diffusive convection under the conditions of thermal radiation and an induced magnetic flux in an asymmetric channel that has a slip boundary has lacked researcher interest until now. The present work is an attempt along these lines. The differential equations are first modeled. The numerical approach is also used to solve highly nonlinear equations. Furthermore, various parameters, such as thermal radiation, solute concentration, velocity, nanoparticle fraction volume, and pressure surge, are evaluated by using graphical representations.

## 2. Mathematical Formulation

### 2.1. Williamson Nanofluid Model

The stress tensor of Williamson fluid model [25] is given as

(1) $$S=-\left[\mu_{\infty }+\left(\mu_{0}+\mu_{\infty }\right){\left(1-\Gamma \dot{\gamma }\right)}^{-1}\right]\dot{\gamma }$$

where $\mu_{0}$ represents the viscosity of zero shear rate, Γ is the time constant, $\mu_{\infty }$ denotes the infinite shear rate viscosity, and $\dot{\gamma }$ is given as

(2) $$\dot{\gamma }=\sqrt{\frac{1}{2}\underset{i}{\sum }\underset{j}{\sum }{\dot{\gamma }}_{ij}{\dot{\gamma }}_{ji}}=\sqrt{\frac{1}{2}\Pi },\;$$

where Π (the invariant second strain tensor) $=\frac{1}{2}$ trace ${\left(\nabla V+{\left(\nabla V\right)}^{*}\right)}^{2},$ and ∗ represents the transpose.

Because Equation (1) is assessing when $\Gamma \dot{\gamma }<1$ and $\mu_{\infty }=0$, it is necessary to redefine the stress tensor as

(3) $$S=-\mu_{0}\left[{\left(1-\Gamma \dot{\gamma }\right)}^{-1}\right]\dot{\gamma }=-\mu_{0}\left[\left(1+\Gamma \dot{\gamma }\right)\right]\dot{\gamma }$$

### 2.2. Formulation

Williamson nanofluid peristaltic flow has been examined under incompressible, electrically conducting conditions confined in a channel with a width of $r_{3}+r_{2}$. The sinusoidal wave train moves with uniform speed at the channel walls, creating a source of flow. The system rectangular coordinates are drawn while keeping the center line of the channel at the X-axis and Y-axis on the cross section. The channel left wall is conserved at solvent concentration $C_{1}$, temperature $T_{1}$, and nanoparticle concentration $\Theta_{1}$. On the other hand, the right wall is conserved at solute concentration $C_{0}$, temperature $T_{0}$, and nanoparticle concentration $\Theta_{0}$. The velocity under two-directional and two-dimensional flow is V=(U(X,Y,t),V(X,Y,t),0). Moreover, an outer transverse uniformly magnetic field is taken as ${\hat{H}}_{0}$ and the induced magnetic field as ${\hat{H}}_{1}^{+}\left(h_{X}\left(X,Y,t\right),H_{0}+h_{Y}\left(X,Y,t\right),0\right)$, and the cumulative magnetic force is calculated as ${\hat{H}}^{+}\left(h_{X}\left(X,Y,t\right),H_{0}+h_{Y}\left(X,Y,t\right),0\right)$.

The geometric description of a wall’s shape is defined as follows [25,41]:

(4) $$Y=H_{1}=r_{3}+r_{4}\cos[\frac{2\pi }{\lambda }\left(X-ct\right)],\;Y=H_{2}=-r_{2}-r_{1}\cos[\frac{2\pi }{\lambda }\left(X-ct\right)+\beta ]$$

where $r_{3}+r_{2}$ stands for the width of channel, λ represents the wavelength, $(r_{4},$ $r_{1})$ is the wave amplitudes, t denotes the time, and c represents the velocity speed. The phase difference β ranges from 0≤β≤π, where β=0 is the symmetric channel without a phase wave and β=π defines a channel with a phase wave. Additionally, $r_{3},$ $r_{1},$ $r_{2},$ $r_{4}$ and β satisfy the condition $r_{4}^{2}+r_{1}^{2}+2r_{4}r_{1}\cos\beta \leq {\left(r_{3}+r_{2}\right)}^{2}$.

The governing equations in component forms that are relevant to the issue under discussion are as follows:

(a) The equation of continuity is defined as

(5) $$\frac{\partial U}{\partial X}+\frac{\partial V}{\partial Y}=0$$

(b) When induced magnetic fields [17] and mixed convection [50] are taken into consideration, the component version of the momentum equation is defined as

(6) $$\begin{array}{c} \rho_{f}\left(\frac{\partial }{\partial \overset{¯}{t}}+U\frac{\partial }{\partial X}+V\frac{\partial }{\partial Y}\right)U\\ \;\;\;\;\;\;\;\;\;\;\;\;\;\;\;\;\;\;=-\frac{\partial p}{\partial X}-\frac{\partial S_{XX}}{\partial X}-\frac{\partial S_{XY}}{\partial Y}-\frac{\mu_{e}}{2}\left(\frac{\partial H^{{+}^{2}}}{\partial X}\right)+\mu_{e}\left(h_{X}\frac{\partial h_{X}}{\partial X}+h_{Y}\frac{\partial h_{X}}{\partial Y}+H_{0}\frac{\partial h_{X}}{\partial Y}\right)\\ \;\;\;\;\;\;\;\;\;\;\;\;\;\;\;\;\;\;+g\{\left(1-\Theta_{0}\right)\rho_{f0}\;\;\left\{\beta_{T}\left(T-T_{0}\right)+\beta_{C}\left(C-C_{0}\right)\right\}-\left(\rho_{p}-\rho_{f0}\right)\left(\Theta -\Theta_{0}\right)\},\; \end{array}$$

(7) $$\rho_{f}\left(\frac{\partial }{\partial \overset{¯}{t}}+U\frac{\partial }{\partial X}+V\frac{\partial }{\partial Y}\right)V=-\frac{\partial p}{\partial Y}-\frac{\partial S_{YX}}{\partial X}-\frac{\partial S_{YY}}{\partial Y}-\frac{\mu_{e}}{2}\left(\frac{\partial H^{{+}^{2}}}{\partial Y}\right)+\mu_{e}\left(h_{X}\frac{\partial h_{Y}}{\partial X}+h_{Y}\frac{\partial h_{Y}}{\partial Y}+H_{0}\frac{\partial h_{Y}}{\partial Y}\right)$$

where $\beta_{C},$ $\rho_{f},$ g, $\Theta ,\;\rho_{p},$ $\beta_{T},$ C, $\rho_{f_{0}},$ T, and $\mu_{e}$ denote the fluid’s volumetric solutal expansion factor, fluid density, acceleration, nanoparticle volume fraction, nanoparticle mass density, fluid’s volumetric thermal expansion index, solutal concentration, fluid density at $T_{0}$, temperature, and magnetic permeability, respectively. Moreover $S_{XX},$ $S_{XY},$ and $S_{YY}$ stand for the stresses of the Williamson fluid model expressed in component form. These stresses are obtained from Equation (3) and are specified as [25]

(8) $$S_{XX}=-2\mu_{0}\left(1+\Gamma \dot{\gamma }\right)\frac{\partial U}{\partial X},$$

(9) $$S_{XY}=-\mu_{0}\left(1+\Gamma \dot{\gamma }\right)\left(\frac{\partial U}{\partial Y}+\frac{\partial V}{\partial X}\right)$$

(10) $$S_{YY}=-2\mu_{0}\left(1+\Gamma \dot{\gamma }\right)\frac{\partial V}{\partial Y}$$

(11) $$\dot{\gamma }=2{\left(\frac{\partial U}{\partial X}\right)}^{2}+{\left(\frac{\partial U}{\partial Y}+\frac{\partial V}{\partial X}\right)}^{2}+2\frac{\partial V}{\partial Y}\;\;$$

(c) The thermal energy, which includes viscous dissipation, thermal radiation effects, nanoparticle fraction, and solute concentration, is defined as [50]

(12) $$\begin{array}{c} {\left(\rho c\right)}_{f}\left(\frac{\partial }{\partial t}+U\frac{\partial }{\partial X}+V\frac{\partial }{\partial Y}\right)T\\ \;\;\;\;\;\;\;\;\;\;\;\;\;\;\;\;\;\;=k\left(\frac{\partial^{2}T}{\partial X^{2}}+\frac{\partial^{2}T}{\partial Y^{2}}\right)+{\left(\rho c\right)}_{p}\{D_{B}\left(\frac{\partial \Theta }{\partial X}\frac{\partial T}{\partial X}+\frac{\partial \Theta }{\partial Y}\frac{\partial T}{\partial Y}\right)\;\left(\frac{D_{T}}{T_{0}}\right)\left[{\left(\frac{\partial T}{\partial X}\right)}^{2}+{\left(\frac{\partial T}{\partial Y}\right)}^{2}\right]\}+D_{TC}\left(\frac{\partial^{2}C}{\partial X^{2}}+\frac{\partial^{2}C}{\partial Y^{2}}\right)\\ \;\;\;\;\;\;\;\;\;\;\;\;\;\;\;\;\;\;-\frac{\partial q_{r}}{\partial Y}+\left(S_{XX}\frac{\partial U}{\partial X}+S_{XY}\left(\frac{\partial U}{\partial Y}+\frac{\partial V}{\partial X}\right)+S_{YY}\frac{\partial V}{\partial Y}\right) \end{array}$$

(13) $$\left(\frac{\partial }{\partial t}+U\frac{\partial }{\partial X}+V\frac{\partial }{\partial Y}\right)C=D_{s}\left(\frac{\partial^{2}C}{\partial X^{2}}+\frac{\partial^{2}C}{\partial Y^{2}}\right)+D_{TC}\left(\frac{\partial^{2}T}{\partial X^{2}}+\frac{\partial^{2}T}{\partial Y^{2}}\right)$$

(14) $$\left(\frac{\partial }{\partial t}+U\frac{\partial }{\partial X}+V\frac{\partial }{\partial Y}\right)\Theta =D_{B}\left(\frac{\partial^{2}\Theta }{\partial X^{2}}+\frac{\partial^{2}\Theta }{\partial Y^{2}}\right)+\left(\frac{D_{T}}{T_{0}}\right)\left(\frac{\partial^{2}T}{\partial X^{2}}+\frac{\partial^{2}T}{\partial Y^{2}}\right)$$

where $D_{B},$ ${\left(\rho c\right)}_{p},$ k, ${\left(\rho c\right)}_{f},$ $D_{CT},$ $D_{T},$ $D_{TC},$ and $D_{s}$ represent the coefficient of Brownian diffusion, nanoparticle effective heat capacity, thermal conductivity, fluid heat capacity, Soret diffusivity, the thermophoretic diffusion coefficient, Dufour diffusivity, and solutal diffusivity, respectively. The $q_{r}$ stands for radiative flux for radiation, and it is calculated by using the Rosseland diffusion estimation:

(15) $$q_{r}=-\frac{4\sigma^{*}}{3k^{*}}\frac{\partial T^{4}}{\partial Y}$$

In this study, the immense radiation limit is considered. If there are only very slight temperature variations within the flow path, then the Taylor expansion can be used to adjust $T^{4}$ so that it can be represented as a linear function of temperature. Now, Taylor expansion on $T^{4}\;$ about $T_{0}$ can be defined as

(16) $$T^{4}=T_{0}^{4}+4T_{0}^{3}\left(T-T_{0}\right)+6T_{0}^{2}{\left(T-T_{0}\right)}^{2}+\ldots$$

By omitting the higher powers of T(higher than first) in $\left(T-T_{0}\right),$ we obtain

(17) $$T^{4}=4T_{0}^{3}T-3T_{0}^{4}$$

From Equations (15) and (17), we obtain

(18) $$q_{r}=-\frac{16\sigma^{*}T_{0}^{3}}{3k^{*}}\frac{\partial T}{\partial Y}$$

(19) $$\frac{\partial q_{r}}{\partial Y}=-\frac{16\sigma^{*}T_{0}^{3}}{3k^{*}}\frac{\partial^{2}T}{\partial Y^{2}}$$

where $\sigma^{*}$ represents the Stefan-Boltzmann constant and $k^{*}$ denotes the Rosseland mean absorption.

In the laboratory frame (X,Y), flow is unsteady, but motion is constant in the coordinate system (x,y). When two reference frames are used, the Galilean transformation is represented by

(20) p(x,y)=P(X,Y,t), x=X−ct, u=U−c, y=Y, v=V

Define

$$\overset{¯}{x}=\frac{x}{\lambda },\;a=\frac{r_{4}}{r_{3}},\;\overset{¯}{y}=\frac{y}{r_{3}},\;\delta =\frac{r_{3}}{\lambda },\;\overset{¯}{u}=\frac{u}{c},\;d=\frac{r_{2}}{r_{3}},\;\overset{¯}{t}=\frac{ct}{\lambda },\;h_{2}=\frac{H_{2}}{r_{2}},\;h_{1}=\frac{H_{1}}{r_{3}},\;b=\frac{r_{1}}{r_{3}},\;\overset{¯}{p}=\frac{r_{3}^{2}p}{\mu_{0}c\lambda },\;$$

Pr=(ρc)f υk, Re=ρfcr3μ0, υ=μρf, Le=υDs, Ln=υDB, pm=p+12ReδμeH˜+μρfc2,u=∂Ψ∂y, 

$$v=-\delta \frac{\partial \Psi }{\partial x},\;h_{x}=\frac{\partial \Phi }{\partial y},\;h_{y}=-\delta \frac{\partial \Phi }{\partial x},R_{m}=\sigma \mu_{e}r_{3}c,$$

S1=H0cμeρ, M2=ReRmS12, θ=T−T0T1−T0, 

$$\Omega =\frac{\Theta -\Theta_{0}}{\Theta_{1}-\Theta_{0}},\;\gamma =\frac{C-C_{0}}{C_{1}-C_{0}},\;\overset{¯}{v}=\frac{v}{c},G_{rc}=\frac{g\left(1-\Theta_{0}\right)\rho_{f}\beta_{c}\left(C_{1}-C_{0}\right)r_{3}^{2}}{\mu_{0}c},\;$$

$$G_{rF}=\frac{g\left(\rho_{p}-\rho_{f}\right)\left(\Theta_{1}-\Theta_{0}\right)r_{3}^{2}}{\mu_{0}c},\;Rd=-\frac{16\sigma^{*}T_{0}^{3}}{3k^{*}},We=\frac{c\Gamma }{r_{3}},\;{\overset{¯}{S}}_{xx}=\frac{S_{xx}\lambda }{\mu_{0}c},{\overset{¯}{S}}_{xy}=\frac{S_{xy}r_{3}}{\mu_{0}c},$$

$${\overset{¯}{S}}_{yy}=\frac{S_{yy}r_{3}}{\mu_{0}c},\;N_{b}=\frac{{\left(\rho c\right)}_{p}D_{B}\left(\Theta_{1}-\Theta_{0}\right)}{k},G_{rt}=\frac{gr_{3}^{2}\left(1-\Theta_{0}\right)\left(T_{1}-T_{0}\right)\rho_{f}\beta_{T}}{\mu_{0}c},\;$$

(21) $$N_{TC}=\frac{D_{CT}\left(C_{1}-C_{0}\right)}{k\left(T_{1}-T_{0}\right)},N_{CT}=\frac{D_{CT}\left(T_{1}-T_{0}\right)}{D_{s}\left(C_{1}-C_{0}\right)},\;N_{t}=\frac{{\left(\rho c\right)}_{p}D_{T}\left(T_{1}-T_{0}\right)}{T_{0}k},\;Br=EcPr.\;$$

The dimensionless forms of the abovementioned equations in terms of ψ (the stream function) and Φ (the magnetic force) are

(22) Reδ(ψyψxy−ψxψyy)                 =−∂pm∂x−δ2∂Sxx∂x−∂Sxy∂y−ReS12Φyy−ReS12δ(ΦyΦxy−ΦxΦyy)+Grtθ+Grcγ−GrFΩ,

(23) Reδ3(ψxψxy−ψyψxx)=−∂pm∂y−δ2∂Sxy∂x−δ∂Syy∂y+Reδ2S12Φyy−ReS12δ3(ΦyΦxx−ΦxΦxy),

(24) Reδ(ψyθx−ψxθy)                =1Pr(θyy+δ2θxx)+NTC(δ2γxx+γyy)+Rd θyy                +Ec(δ2Sxxψxy+Sxy(ψyy−δ2ψxx)−δSyyψxy)+Nb(δ2θxΩx+θyΩy)                +Nt(δ2(θx)2+(θy)2),

(25) ReδLe(ψyγx−ψxγy)=(δ2γxx+γyy)+NCT(δ2θxx+θyy),

(26) ReδLn(ψyΩx−ψxΩy)=(δ2Ωxx+Ωyy)+NtNb(δ2θxx+θyy),

(27) $$\psi_{y}-\delta \left(\psi_{y}\Phi_{x}-\psi_{x}\Phi_{y}\right)+\frac{1}{R_{m}}\left(\Phi_{yy}+\delta^{2}\Phi_{xx}\right)=E$$

The nondimensional form of the components of stresses are

$$S_{xx}=-2\left(1+\mathrm{We}\dot{\gamma }\right)\frac{\partial^{2}\psi }{\partial x\partial y},$$

$$S_{xy}=-\left(1+\mathrm{We}\dot{\gamma }\right)\left(\frac{\partial^{2}\psi }{\partial y^{2}}-\delta^{2}\frac{\partial^{2}\psi }{\partial x^{2}}\right),$$

$$S_{yy}=2\delta \left(1+\mathrm{We}\dot{\gamma }\right)\frac{\partial^{2}\psi }{\partial x\partial y},$$

(28) $$\dot{\gamma }={\left[2\delta^{2}{\left(\frac{\partial^{2}\psi }{\partial x\partial y}\right)}^{2}+{\left(\frac{\partial^{2}\psi }{\partial y^{2}}-\delta^{2}\frac{\partial^{2}\psi }{\partial x^{2}}\right)}^{2}+2\delta^{2}{\left(\frac{\partial^{2}\psi }{\partial x\partial y}\right)}^{2}\right]}^{1/2}$$

When the limitations of δ<<1 (long wavelength) and the low Reynolds number are applied, the governing Equations (22)–(28) take on the following forms:

(29) ∂p∂x=∂∂y[(1+We∂2ψ∂y2)(∂2ψ∂y2)]+ReS12Φyy+Grtθ+Grcγ−GrFΩ,

(30) $$-\frac{\partial p}{\partial y}=0,$$

(31) $$\frac{\partial^{2}\theta }{\partial y^{2}}+N_{TC}Pr\frac{\partial^{2}\gamma }{\partial y^{2}}+N_{b}Pr\left(\frac{\partial \theta }{\partial y}\frac{\partial \Omega }{\partial y}\right)+N_{t}Pr{\left(\frac{\partial \theta }{\partial y}\right)}^{2}+Rd\;Pr\frac{\partial^{2}\theta }{\partial y^{2}}+Br\left({\left(\frac{\partial^{2}\psi }{\partial y^{2}}\right)}^{2}+We{\left(\frac{\partial^{2}\psi }{\partial y^{2}}\right)}^{3}\right)=0,$$

(32) $$\frac{\partial^{2}\gamma }{\partial y^{2}}+N_{CT}\frac{\partial^{2}\theta }{\partial y^{2}}=0,$$

(33) $$\frac{\partial^{2}\Omega }{\partial y^{2}}+\frac{N_{t}}{N_{b}}\frac{\partial^{2}\theta }{\partial y^{2}}=0,$$

(34) $$\Phi_{yy}=R_{m}\left(E-\frac{\partial \psi }{\partial y}\right),\;$$

where $G_{rc},$ $N_{TC},$ Re, γ, Ω, Le, $G_{rF},$ Ln, θ, We, $N_{t},$ δ, $N_{b},$ Pr, $N_{CT},$ ψ, the $G_{rt}$ stand for the solutal Grashof number, Dufour parameter, Reynolds number, solutal concentration, nanoparticle volume fraction, Lewis number, nanoparticle Grashof number, nanofluid Lewis number, temperature, Weissenberg number, thermophoresis parameter, wave number, Brownian motion parameter, Prandtl number, Soret parameter, stream function, and thermal Grashof number, respectively.

After reducing pressure from Equations (29) and (30), the stream function (ψ) equation is

(35) ∂2∂y2[(1+We∂2ψ∂y2)(∂2ψ∂y2)]−ReS12Rm∂2ψ∂y2+Grt∂θ∂y+Grc∂γ∂y−GrF∂Ω∂y=0,

In nondimensional form, the mean flow (Q) is derived as

(36) Q=1+F+d, 

where

(37) $$F=\int_{h_{2}\left(x\right)}^{h_{1}\left(x\right)}\frac{\partial \psi }{\partial y}dy=\psi \left(h_{1}\left(x\right)-h_{2}\left(x\right)\right),\;$$

where

(38) $$h_{1}\left(x\right)=1+a\cos2\pi x,{\;h}_{2}\left(x\right)=-d-b\cos(2\pi x+\beta ).\;$$

In order to solve PDEs (29) and (31)–(35) in nondimensional form, the current system must be under the following boundary conditions:

$$\psi =\frac{F}{2},\;\frac{\partial \psi }{\partial y}=-\xi_{1}S_{xy}-1\;\mathrm{on}\;y=h_{1}\left(x\right),$$

(39) $$\psi =-\frac{F}{2},\;\frac{\partial \psi }{\partial y}=\xi_{1}S_{xy}-1\;\mathrm{on}\;y=h_{2}\left(x\right).$$

$$+\xi_{2}\frac{\partial \theta }{\partial y}=0,\;\mathrm{on}\;y=h_{1},$$

(40) $$\theta -\xi_{2}\frac{\partial \theta }{\partial y}=1,\;\mathrm{on}\;y=h_{2},\;$$

$$\gamma +\xi_{3}\frac{\partial \gamma }{\partial y}=0,\;\mathrm{on}\;y=h_{1},$$

(41) $$-\xi_{3}\frac{\partial \gamma }{\partial y}=1,\;\mathrm{on}\;y=h_{2},$$

$$\Omega +\xi_{4}\frac{\partial \Omega }{\partial y}=0,\;\mathrm{on}\;y=h_{1},$$

(42) $$\Omega -\xi_{4}\frac{\partial \Omega }{\partial y}=1,\;\mathrm{on}\;y=h_{2},\;$$

(43) $$\Phi =0\;\mathrm{at}\;y=h_{1}\left(x\right)\;\mathrm{and}\;y=h_{2}\left(x\right).\;$$

The no-slip conditions are represented when $\xi_{1},$ $\xi_{2},$ $\xi_{3},$ $\xi_{4}=0$ under the aforementioned conditions.

## 3. Numerical Simulation and Graphical Solutions

The solutions to Equations (31)–(35) and (29) can be found via numerical simulations. The numerical simulations to Equations (31)–(35) and (29) are computed by using Mathematica software (Mathematica 13, 2021, Wolfram, Oxfordshire, UK). NDSolve, a built-in program in Mathematica, is used to solve equations. This command uses interpolating function objects to iteratively solve problems. To highlight the flow thermodynamics characteristics, a numerical approach to solutions is applied to generate a graphic analysis of numerous model parameters.

**Figure 1:**
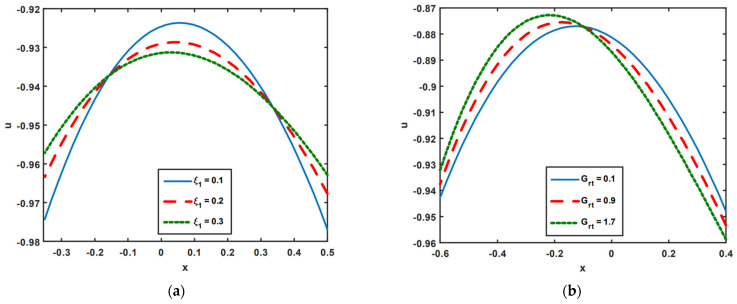

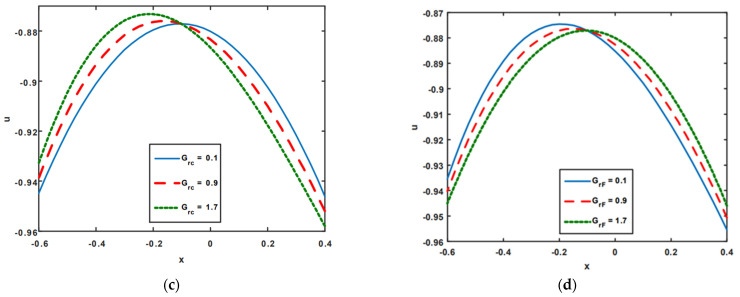
(**a**–**d**) Velocity impact on $\xi_{1}$, $G_{rt}$, $G_{rc}$, and $G_{rF}$.

Figure 1a–d are composed to investigate the interactions of flowing fluid on velocity slip $\xi_{1}$, the thermal Grashof number $G_{rt}$, the solutal Grashof number $G_{rc}$, and the nanoparticle Grashof number $G_{rF}.$ Figure 1a clearly illustrates that the magnitude value of fluid velocity increases at the channel’s center y∈[−0.25,0.35], owing to the growing impact of velocity slip $\xi_{1}.$ This is due to the reduction in resistance caused by slip. However, at wall ends y∈[−0.35,−0.25] and y∈[0.35,0.5], the opposite result is observed. Here, the magnitude of fluid velocity drops as $\xi_{1}$ increases. Figure 1b,c emphasizes the outcomes of fluid velocity on the thermal Grashof number $G_{rt}$ and the solutal Grashof number $G_{rc}$. Figure 1b,c demonstrates that as $G_{rt}$ and $G_{rc}$ increase, the magnitude value of fluid velocity falls when traveling toward the left wall y∈[−0.6,−0.1], but the reverse impact is observed when traveling toward the right wall y∈[−0.1,0.4]. Figure 1d shows that opposing scenarios arise in the case of $G_{rF}$ when compared to $G_{rt}$ and $G_{rc}$. Here, the magnitude value of fluid velocity enhances as it approaches the left wall at y∈[−0.6,−0.1], whereas it drops as it proceeds toward the right wall at y∈[−0.1,0.4] thanks to the rising impacts of $G_{rc}$.

**Figure 2:**
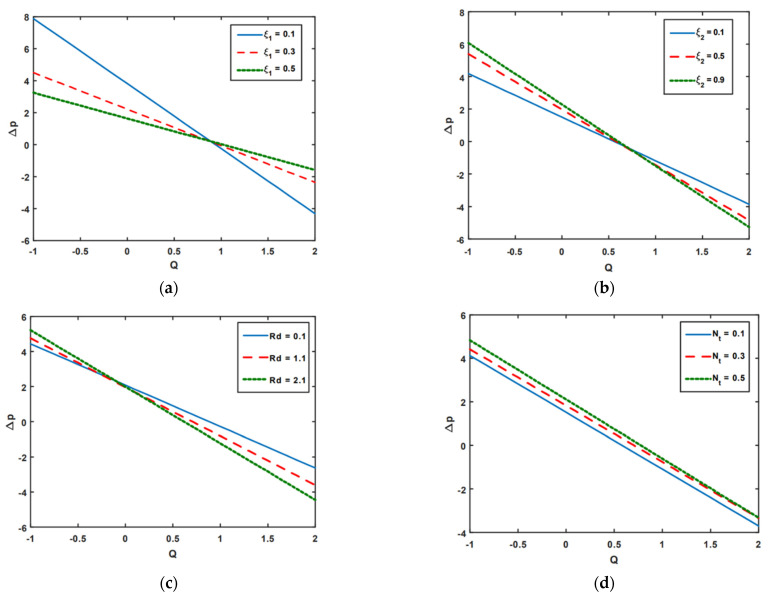
(**a**–**d**) Pressure rising impact on $\xi_{1}$, $\xi_{2}$, Rd, and $N_{t}$.

Figure 2a–d illustrates the impact of pressure rises on velocity slip $\xi_{1},$ thermal slip $\xi_{2},$ thermal radiation Rd, and thermophoresis $N_{t}.$ Pumping zones are categorized into the following groups to examine the feature of pressure rise: (a) the region of the peristaltic (Q>0,Δp>0), where peristalsis waves regulate pressure and move fluid along its track of propagation; (b) the augmented (Q>0,Δp<0) zone, where peristaltic force-induced pressure increases the flow; (c) the region of retrograde (Q<0,Δp>0), where peristalsis is resisting the flow; and (d) free (Δp=0) pumping regions. The only source of flow in this area is provided by the peristalsis walls. As seen in Figure 2a, the pressure rise tends to drop in the retrograde; the velocity slip parameter $\xi_{1}$ increases in the peristaltic regions; and a pressure rise increases in the augmented area. It is noted in Figure 2b that when temperature slip parameter $\xi_{2}$ increases, pressure rising increases in the retrograde, free, and peristaltic zones. Moreover, it decreases in the augmented region. Furthermore, Figure 2c demonstrates that Rd behaves in a similar manner to $\xi_{2}.$ According to Figure 2c, because of the increasing impact of thermal radiation Rd, the pressure rising increases in the retrograde region, while in the augmented region, peristaltic region, and free pumping zones, the pressure rising decreases owing to the increasing impact of thermal radiation. According to an analysis of Figure 2c, boosting the $N_{t}$ parameters results in a rise in pressure in all peristaltic zones.

**Figure 3:**
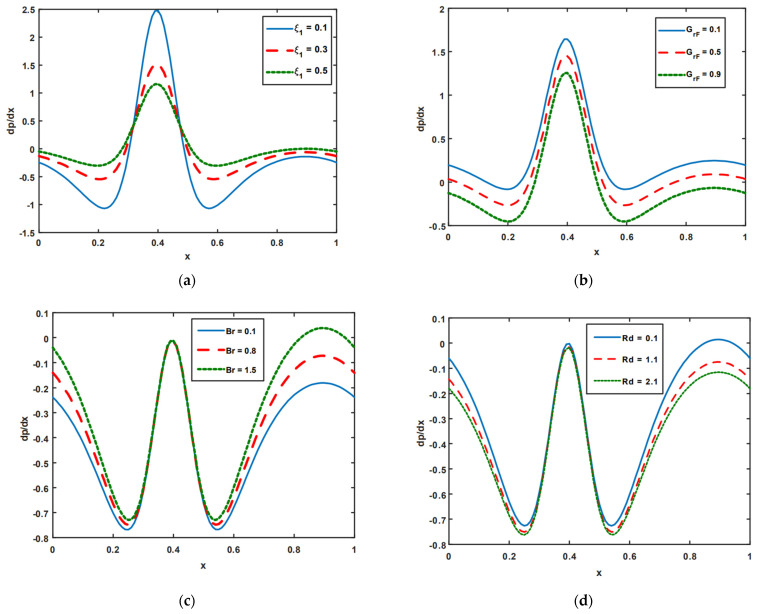
(**a**–**d**) Pressure gradient impact on $\xi_{1},$ $G_{rF},$ Br, and Rd.

Figure 3a–d is plotted to examine the impact of the pressure gradient on velocity slip $\xi_{1}$, the nanoparticle Grashof number $G_{rF}$, the Brinkman number Br, and thermal radiation Rd. From Figure 3a, it is noted that with an increase in the velocity slip parameter $\xi_{1}$ the pressure gradient drops when y∈[0.3,0.5], while in the regions y∈[0.0,0.3] and y∈[0.5,0.1], the opposite effects are noted. In these regions, pressure gradients tend to rise. The pressure gradient tends to fall with the increasing behavior of $G_{rF}$ (see Figure 3b). Figure 3c is shown to explore the impacts of Br on the pressure gradient. It is depicted in Figure 3c that the magnitude value of the pressure gradient decreases when y∈[0.0,0.25] and y∈[0.5,1.0], with an increase in Br values, while there is no variation in the pressure gradient when y∈[0.25,0.5]. From Figure 3d, it is observed that heat radiation Rd has the opposite effect to that of Br.

**Figure 4:**
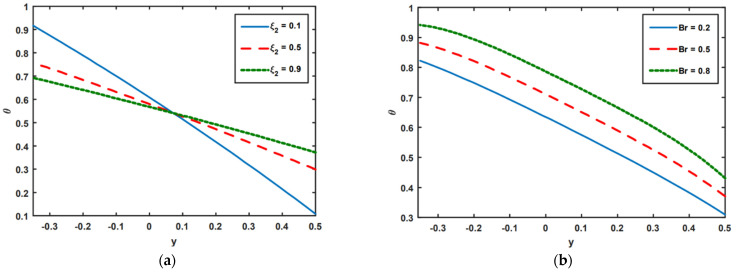

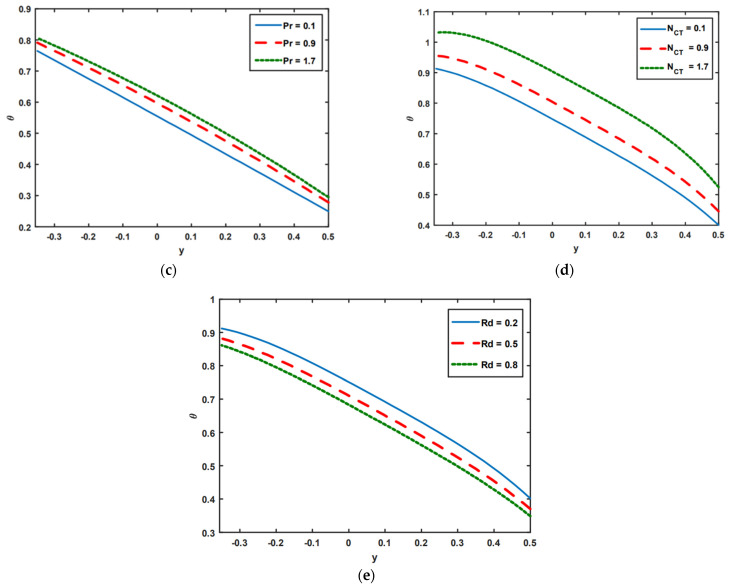
(**a**–**e**) Impact of $\xi_{2},$ Br, Pr, $N_{CT},$ and Rd on temperature profile.

Figure 4a–e is presented to look into the effects of temperature on thermal slip $\xi_{2},$ the Brinkman number Br, the Prandtl number Pr, Soret $N_{CT},$ and thermal radiation Rd. It can be noticed that the temperature is reduced in the region y∈[−0.35,0.1] thanks to the increasing thermal slip $\xi_{2}$ parameter; however, opposite effects are observed in the region y∈[0.1,0.5]. Here, the temperature rises as the thermal slip parameter $\xi_{2}$ is increased. Figure 4b–d is presented to examine the impact of Br, Pr, and $N_{CT}$. It is seen from Figure 4b–d that temperature tends to rise as the Br, Pr, and $N_{CT}$ values grow. Br signifies the impact of viscous dissipation, which raises the heat transfer rate. Physically, low Prandtl numbers indicate strong thermal diffusivity, whereas a high Prandtl number reveals progressive momentum. It is noteworthy that in the case of Rd, the results are the opposite. As heat radiation increases, temperatures tend to drop (see Figure 4e). This is because heat radiation and thermal conduction are inversely related. This indicates that the heat radiation in the system is at its maximum, reducing the fluid’s capacity to conduct heat.

**Figure 5:**
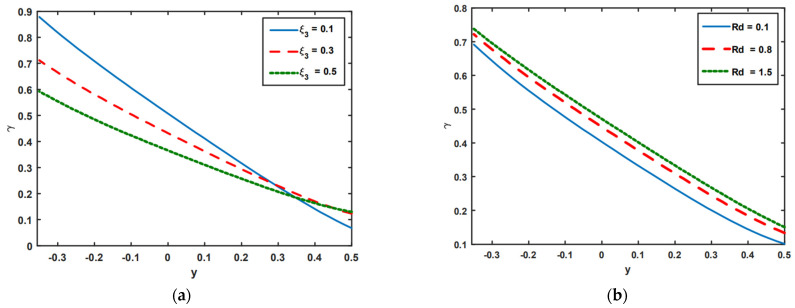

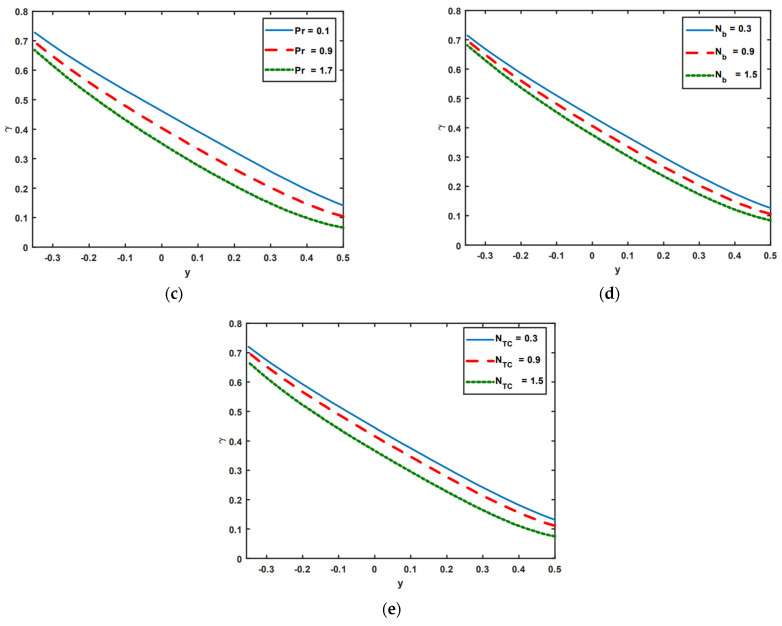
(**a**–**e**) Impact of $\xi_{3},$ Rd, *Pr*, $N_{b},$ and $N_{TC}$ on concentration profile.

Figure 5a–e is presented to observe the impact of the slip parameter of concentration $\xi_{3},$ thermal radiation Rd, Prandtl number *Pr*, Brownian motion $N_{b},$ and Dufour number $N_{TC}$ on the concentration profile. It is seen in Figure 5a that because of the increasing impact of concentration slip $\xi_{3}$, the concentration profile decreases. The profile of concentration is improved by the rising behavior of thermal radiation Rd (see Figure 5b). Figure 5c–e is presented to examine the effects of *Pr*, $N_{b}$, and $N_{TC}$ on the concentration profiles. It is noted from these figures that the concentration profile tends to drop when the influence of *Pr*, $N_{b}$, and $N_{TC}$ increases. This occurs thanks to a substantial nanoparticle transition from a hot to a cold region, which narrows the concentration distribution.

**Figure 6:**
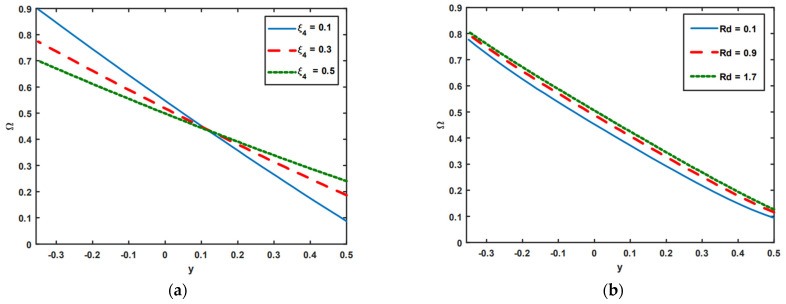

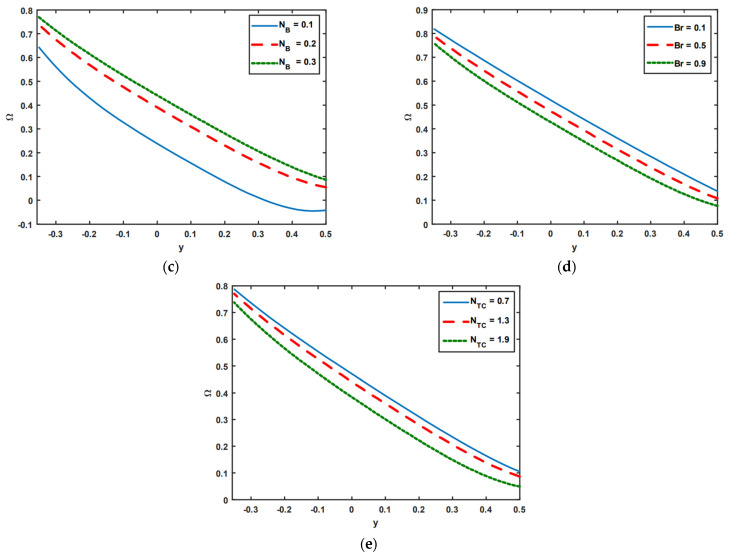
(**a**–**e**) Impact of $\xi_{4},$ Rd, $N_{b},$ Br, and $N_{TC}$ on nanoparticle fraction.

Figure 6a–e illustrates the impact of the slip parameter of nanoparticle fraction $\xi_{4},$ thermal radiation Rd, Brownian motion $N_{b}$, Brinkman number Br, and Dufour number $N_{TC}$ on the profile of nanoparticle fraction. Figure 6a highlights the impact of the slip parameters of nanoparticle fraction $\xi_{4}$. It is noted in Figure 6a that in region y∈[−0.35,0.1], the nanoparticle fraction decreases with an increasing slip parameter of nanoparticle fraction $\xi_{4},$ whereas in the domain y∈[0.1,0.5], the opposite effects are found. Here, nanoparticle fraction increases with an increase in the values of the slip parameter of nanoparticle fraction $\xi_{4}.$ Figure 6b,c shows the effects of thermal radiation Rd and Brownian motion $N_{b}$ on nanoparticle fraction. It is shown in Figure 6b,c that the profile of the nanoparticle fraction rises as a result of the increasing behavior of thermal radiation Rd and Brownian motion $N_{b}.$ Nanofluids have an extensive heat flux, which might alter the system distribution. Moreover, the opposite results are observed in the cases of Br and $N_{TC}.$ In these cases, the profile of the nanoparticle tends to reduce because of the growing influence of Br and $N_{TC}$ (see Figure 6d,e).

**Figure 7:**
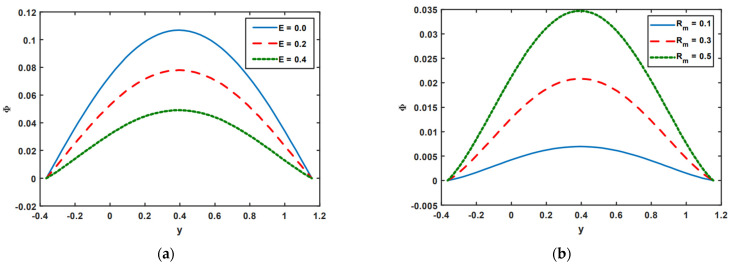
(**a**,**b**) Impact of E and $R_{m}$ on magnetic force function Φ.

Figure 7a,b shows the consequences of the magnetic force function on electric field E and magnetic Reynold number $R_{m}$. Figure 7a,b also demonstrates the implications of the magnetic force function on electric field E and magnetic Reynolds number $R_{m}$. According to Figure 7a, the rising influence of electric field E causes the strength of the magnetic force function Φ to decline. The opposite effects are noted for the case of the magnetic Reynolds number $R_{m}.$ As the impact of the magnetic Reynolds number $R_{m}$ rises, it is seen in Figure 7b that the magnetic force function increases.

**Figure 8:**
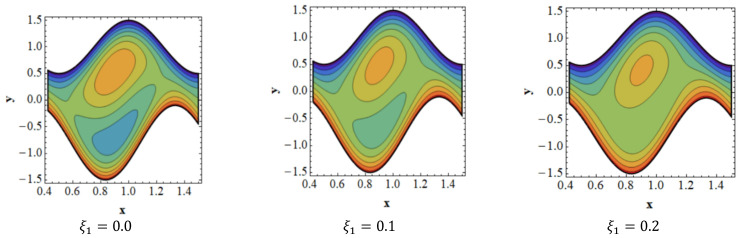
Consistency of streamlines for values of $\xi_{1}$.

**Figure 9:**
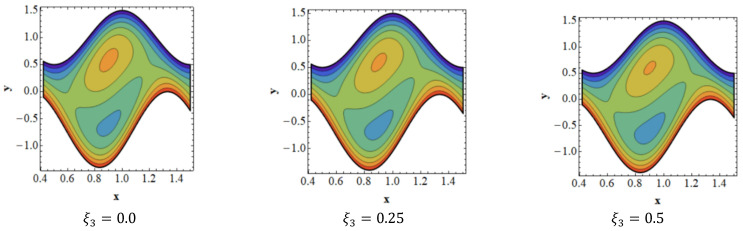
Consistency of streamlines for values of $\xi_{3}$.

**Figure 10:**
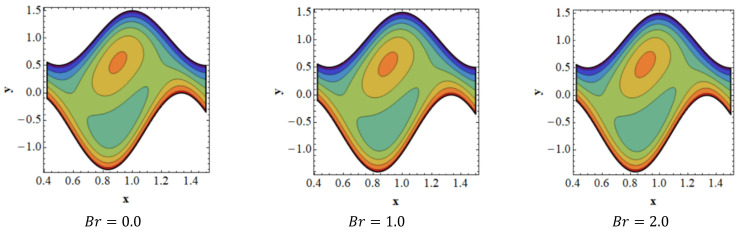
Consistency of streamlines for values of Br.

**Figure 11:**
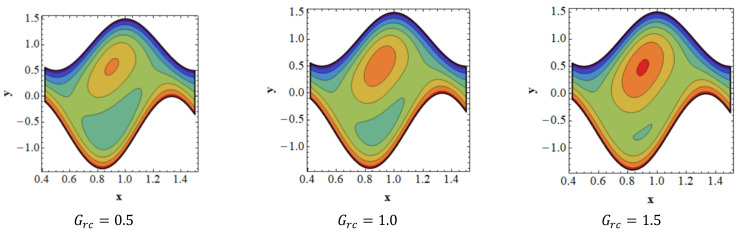
Consistency of streamlines for values of $G_{rc}$.

Trapping is a crucial topic in peristaltic flow. It can be identified by the existence of an inner fluid mass that is in motion and is enclosed by streamlines with a peristaltic pattern. Streamlines capture the fluid mass bolus and use peristaltic waves with a lot of obstructions and high pressure to move it forward. Figure 8 depicts the impact of velocity slip $\xi_{1}$ on streamlines. Figure 8 shows that when the influence of the velocity slip $\xi_{1}$ increases, the number of trapped boluses in the lower portion of the channel decreases, while the size of the trapping bolus decreases in the top portion. Figure 9 is presented to demonstrate the effect of streamlines on concentration slip $\xi_{3}$. It is shown in Figure 9 that the size of the trapping bolus grows in the bottom portion of the channel, but opposite effects are observed in the upper portion of the channel thanks to an increase in the behavior of concentration slip $\xi_{3}$. Consistency among streamlines for increasing values of Br is shown in Figure 10. Thanks to the growing influence of Br, it can be seen in Figure 10 that the trapped bolus’ size remains unchanged in the lower part of the channel, but only slightly changes in the trapped bolus occur in upper part of channel. The impacts of streamlines on various values of $G_{rc}$ are illustrates in Figure 11. As the influence of $G_{rc}$ rises, it can be seen in Figure 11 that the size of the trapped bolus decreases in the bottom portion of the channel, while the number and the size of the trapped bolus increase in the upper region of the channel.

## 4. Conclusions

The prime aim of the present work was to investigate the mathematical model of peristalsis flow of nanofluid by using thermal radiation, an induced magnetic field, double-diffusive convection, and slip boundary conditions in an asymmetric channel. The linear mathematical relationship was used to convert the rheological equations from fixed to wave frames. Afterward, with the aid of dimensionless variables, the rheological equations were transformed into nondimensional forms. Mathematica software was used to evaluate the numerical value of rheological equations. The effect of significant hydromechanical parameters on trapping, velocity, concentration, magnetic force function, nanoparticle volume fraction, temperature, pressure gradient, and pressure rise were visually assessed.

The main interpretations are as follows:

- The rising impact of velocity slip results in a reduction in resistance that increases fluid velocity at the channel’s center.
- Thanks to the increasing impact of thermal radiation, the pressure rising increases in retrograde regions, while in the augmented region, peristaltic region, and free pumping zones, the pressure rising decreases.
- As heat radiation increases, temperatures tend to drop because heat radiation and thermal conduction are inversely related.
- A significant nanoparticle transition from a hot to a cold region causes a decrease in concentration distribution.
- The size of the trapping bolus decreases in the upper portion of the channel, while the quantity of trapped boluses in the lower portion falls as the influence of velocity slip grows.

## Data Availability

Not applicable.
